# Levels of major and trace elements in fennel (*Foeniculum vulgari* Mill.) fruits cultivated in Ethiopia

**DOI:** 10.1186/2193-1801-4-5

**Published:** 2015-01-03

**Authors:** Feleke Demissie Endalamaw, Bhagwan Singh Chandravanshi

**Affiliations:** 4 Department of Chemistry, College of Natural Sciences, Addis Ababa University, P.O. Box 1176, Addis Ababa, Ethiopia; 5 Department of Chemistry, Faculty of Natural and Computational Sciences, Wolayta Sodo University, P.O. Box: 138, Wolayta Sodo, Ethiopia

**Keywords:** Fennel, *Foeniculum vulgari* Mill., *Ensilal*, Trace elements, Spice, Ethiopia

## Abstract

**Background:**

Sweet fennel (*Foeniculum vulgare* Mill.) is one of the precious spices. Almost all parts of fennel plant are edible. The herb is used as carminative, digestive, diuretic, cosmetic and medicine.

**Methods:**

A 0.5 g of the oven dried fennel fruit and soil samples were digested by wet-digestion method. The levels of selected elements (Ca, Mg, Fe, Mn, Cu, Cr, Co, Zn, Ni, Cd and Pb) were determined in sweet fennel fruit (*Foeniculum vulgari* Mill.) and soil from Addis Ababa (Central Ethiopia) and Gojjam (Northern West Ethiopia) by flame atomic absorption spectrometry (FAAS).

**Results:**

The elemental concentrations (μg/g) in fennel fruit were: Ca (20,500–23,000), Mg (1,310–3,460), Fe (1,140–1,900), Mn (31–51), Cu (24–103), Cr (91–98), Co (26–71), Zn (37–45), Ni (19–24), and Cd (1.6–1.9) while in the soil were: Ca (1,440–1,780), Mg (1,260–3,310), Fe (26,900–28,000), Mn (1,460–1,980), Cu (51–101), Cr (127–141), Co (54–143), Zn (99–104), Ni (98–161), and Cd (1.7–2.9). Pb was below the method detection limit in both the fennel fruit and soil.

**Conclusion:**

The Ethiopian fennel fruits are rich in Ca and Mg and other essential elements (Fe, Cu, Co and Zn) and can be used as good supplement for human being in particularly for children and pregnant women. The toxic element Cd is at trace level and Pb is not detected in the fennel fruit. Thus, Ethiopian fennel fruits are safe for human consumption.

## Introduction

Sweet fennel (*Foeniculum vulgare* Mill.) is one of the precious spices. It is a highly aromatic (Parthasarathy et al. 2008) and flavourful herb with culinary, cosmetic (Rasul et al. 2012; Kim et al. 2004) and medicinal uses (Javidnia et al. 2003; He and Huang 2011; Cetin et al. 2010; Faudale et al. 2008; Mohamad et al. 2011; Qiu et al. 2012; Senatore et al. 2013; Shahat et al. 2011; Dadalioglu and Evrendilek 2004). It has also nutritional values (Renna and Gonnella 2012). It belongs to the family Apiaceae (formerly the Umbelliferae) (Qiu et al. 2012). In Ethiopia, the plant is called *Ensilal*. It is native to Southern Europe and the Mediterranean region and is cultivated mainly in India, China, Guatemala, Sri Lanka, Greece, and many other countries in Europe and America (Parthasarathy et al. 2008; He and Huang 2011; Rather et al. 2012; Rahimi and Ardekani 2013; Chéry et al. 2008). As to our knowledge, it is cultivated only in Ethiopia in Africa.

Almost all parts of fennel plant are edible. The herb is used as carminative, digestive, and diuretic. It is used in treating respiratory and gastrointestinal disorders (Diao et al. 2014; El-Motaium and El-Seoud 2007). Furthermore, its insect rippling and antimicrobial properties against a wide range of microorganisms have been well established (Qiu et al. 2012; Oktay et al. 2003). Fennel essential oil is used in cosmetics, pharmaceuticals, and perfumery and as a food additive and flavouring agent in food products (Kim et al. 2004; Mohamad et al. 2011; Rather et al. 2012; Oktay et al. 2003). The fennel fruits are used as flavourings in baked goods, meat and fish dishes, ice cream, alcoholic beverages and herb mixtures. Fennel fruit tea and fennel fruit water are very common traditional medicines for flatulence treatment (He and Huang 2011; Rather et al. 2012). In China, fennel fruit is used both as tradition Chinese medicine and as common food with Chinese name *Xiaohuixiang* (He and Huang 2011).

There are various demands for elemental information in biological and medical samples. Trace elements exhibit significant functions with respect to living organisms in terms of oxygen carrying, electron transfer, enzyme catalyzing, etc. (Singh and Garg 2006; Divrikli et al. 2006; Demirel et al. 2008). For all the elements essential for metabolism, there exists a range of intake over which their supply is adequate for the body beyond which deficiency and toxic effects are observed (Mallinckrodt and Meissne 1994). All the nutrient elements are primarily supplied through diet. However, this may change, depending on age, sex, health status, geographical and climatic conditions (O'Dell and Sunde 1997). Therefore, it is essential to determine elemental contents of food items. WHO and FAO have recommended selective studies of individual foodstuffs as an important step in the estimation of dietary intake of trace elements (FAO/WHO 2001). It is a good practice to include micronutrient density of the diet component in addition to energy and protein adequacy of it (FAO/WHO 2001).

There are some works done on the elemental level of infusion and decoction tea (Desideri et al. 2011; Ozcan et al. 2008; Szymczycha-Madeja et al. 2012; Sembratowicz and Rusinek-prystupa 2014; Başgel and Erdemoğlu 2006; Zengin et al. 2008) of the fennel fruit, steam and leaf. Fennel fruit is eaten cooked or raw in different part of the world. Chewing fennel fruit after a meal is a common practice in India (Shirahatt et al. 2010). Ethiopians use raw or roasted and ground form of it as spice. It is also used to flavor local drinks such as *areke* and *tej*. Moreover, in Ethiopia the fennel fruit is used as a spice in the preparation of *shiro* and *berbere* and the powdered roasted fennel fruit is added in to traditional dish like *doro wot* (Getahun 1976; Zuberi et al. 2014). There is no report on the elemental content of fennel fruit in Ethiopia in any form and only few reports (Khattak and Khattak 2011; Chowdhary et al. 2014; Ozcan and Akbulut 2007; Kumar et al. 2005) are available worldwide on the total elemental content of the fennel fruit. None of them correlate the nutrient found in the fennel fruit with the soil it is grown. This study was therefore; designed to assess the levels of major and trace elements (Ca, Mg, Fe, Mn, Cu, Cr, Co, Zn, Ni, Cd and Pb) in fennel fruit cultivated in the farmland in Ethiopia. To assess the significance of soil to the growth of fennel, it was also analysed for major and trace elements using flame atomic absorption spectrometry.

## Methods

### Instruments and reagents

Blending device (Moulinex, France), digital analytical balance (Adam, Model WL 3000, Switzerland), hot plate, air-circulating oven (Digitheat, J.P. Selecta, Spain) and flame atomic absorption spectrophotometer (FAAS) (Buck Scientific, Model 210VGP AAS, East Norwalk, USA) were used.

Chemicals and reagents that were used in the analysis were all analytical grades. 69–72% HNO_3_ (Supreme Enterprises Cantt, India), 70% HClO_4_ (A.C.S. Reagent, Aldrich, UK) and 36% H_2_O_2_ (Scharlau Chemie, European Union, UN2014) were used for digestion of powdered fennel fruit and soil samples. Lanthanum nitrate hydrate (99.9%, Aldrich, Muwaukee, USA) was used to prevent the chemical interference of phosphate ion on Ca and Mg during the analysis of the samples. Stock standard solution of concentration 1000 mg/L in 2% HNO_3_ of the elements Ca, Mg, Mn, Fe, Cu, Zn, Ni, Co, Cr, Pb and Cd (Buck Scientific Puro-Graphic) standard solutions were used to prepare intermediate standard solutions to obtain calibration curves for the determination of elements in the samples and recovery study.

### Sampling and sample preparation

The study locations were Addis Ababa, Bichena, Debre Markos and Finote Selam. Addis Ababa is the capital city of Ethiopia. Bichena and Debre Markos are located in east Gojjam (Ethiopia); they are 299 km and 265 km from Addis Ababa, respectively. Finote Selam is found in west Gojjam, which is 380 km away from the capital city. Addis Ababa was selected because it is expected to be relatively contaminated area and to see the accumulation effect of the plant. Gojjam was selected because fennel is consumed in these zones every day in different ways, as spice, flavouring agent in local *areki* and used as medicinal plant. Latitude, longitude, altitude, annual average temperature and rainfall of the sampling sites of fennel fruit are given in Table 1.

**Table 1:** **Latitude, longitude, altitude, annual average temperature and annual rain fall of the sites**

Name of sites	Latitude (N)	Longitude (E)	Altitude (m)	Annual average temperature (°C)	Annual average rain fall (mm)
**AA**	8°59′	38°48′	2,354	12-20	1,210
**B**	10°27′	38°12′	2,539	9.8-22	1,250
**DM**	10°21′	37°43′	2,440	14.2-18.6	1,340
**FS**	10°42′	37°16′	1,905	9.8-23.5	1,450

AA = Addis Ababa, B = Bichena, DD = Debre Markos and FS = Finote Selam.

Fennel fruit samples were collected during the dry season (March – April 2010). A total of 5 sub-sites were selected for each site and 100 g fennel fruit and 100 g soil were collected from each sub-site. The sub-sites were selected randomly. The samples collected in each sub-sites were pooled and 0.5 kg of bulk sample were collected for each of the sites. The soil samples were collected at a depth of 20 cm from the surface of the top soil. Samples were sealed in polyethylene bags and transported to the laboratory. All the samples were dried in the oven at 70°C for 48 hours for fennel fruit and 72 hours for soil until the mass of the samples became constant.

### Optimization of digestion procedure

In this study nitric and perchloric acids were used during optimization procedure for fennel fruit and a total volume of acid was taken to be 4 mL. The ratio of the acids, temperature and time of digestion were optimized. The results are given in Table 2. Therefore, the procedure with total of 4 mL reagents volume (2.5 mL HNO_3_ and 1.5 mL HClO_4_), heating at 210°C and 150 min digestion time was selected for this study. Similar optimization also carried out for the soil sample digestion using HNO_3_, HClO_4_ and H_2_O_2_. The results are given in Table 3. The optimum condition obtained was 2 mL HNO_3_ (69–72%), 4 mL HClO_4_ (70%) and 1.5 mL of H_2_O_2_ (36%) and at temperature 270°C for 180 min. The solid dry sample (for both soil and fennel fruit) digested by this procedure was 0.5 g.

**Table 2:** **Reagent types and volumes, temperature and time attempted during optimization of digestion of 0.5 g fennel fruit**

No.	Reagent volume (mL)			Temperature (°C)	Time (min)	Results
	HNO _3_	HClO _4_	Total			
1	1	3	4	270	180	Yellow
2	1.5	2.5	4	270	180	Lightly yellow
3	2	2	4	270	180	Almost clear
**4**	**2.5**	**1.5**	**4**	**270**	**180**	**Clear solution**
5	3	1	4	270	180	Clear solution
6	3.5	0.5	4	270	180	Almost clear
7	2.5	1.5	4	120	180	Yellow
8	2.5	1.5	4	150	180	Lightly yellow
9	2.5	1.5	4	180	180	Almost clear
**10**	**2.5**	**1.5**	**4**	**210**	**180**	**Clear solution**
11	2.5	1.5	4	240	180	Clear solution
12	2.5	1.5	4	270	180	Clear solution
13	2.5	1.5	4	270	105	Light brown with suspension
14	2.5	1.5	4	270	120	Yellow
15	2.5	1.5	4	270	135	Lightly yellow
**16**	**2.5**	**1.5**	**4**	**270**	**150**	**Clear solution**
17	2.5	1.5	4	270	165	Clear solution
18	2.5	1.5	4	270	180	Clear solution

Rows with bold font indicate the optimal condition for the given parameter.

**Table 3:** **Reagent types and volumes, temperature and time attempted during optimization of digestion of 0.5 g soil**

No.	Reagent volume (mL)			Temperature (°C)	Time (min)	Results
	HNO _3_	HClO _4_	H _2_O _2_			
1	2.2	4.4	1	300	180	Deep yellow with suspension
**2**	**2**	**4**	**1.5**	**300**	**180**	**Light yellow with no suspension**
3	1.8	3.6	2	300	180	Light yellow with no suspension
4	2	4	1.5	240	180	Deep yellow with suspension
**5**	**2**	**4**	**1.5**	**270**	**180**	**Light yellow with no suspension**
6	2	4	1.5	300	180	Light yellow with no suspension
7	2	4	1.5	270	120	Deep Yellow with suspension
8	2	4	1.5	270	150	Light yellow with suspension
**9**	**2**	**4**	**1.5**	**270**	**180**	**Light yellow with no suspension**

Rows with bold font indicate the optimal condition for the given parameter.

Fennel fruit samples were digested by applying the optimized procedure. A 0.5 g of well-powdered fennel fruit sample was added into a round bottom flask (100 mL). To this flask 2.5 mL HNO_3_ (69–72%) and 1.5 mL HClO_4_ (70%) were added and the mixtures were digested on a micro Kjeldahl digestion apparatus by setting the temperature at 210°C for 150 min.

Soil samples were digested by applying the optimized procedure. A 0.5 g of well-powdered soil sample was added into a round bottom flask (100 mL). To this flask 2 mL HNO_3_ (69–72%), 4 mL HClO_4_ (70%) and 1.5 mL of H_2_O_2_ (36%) were added and the mixtures were digested on a micro Kjeldahl digestion apparatus by setting the temperature at 270°C for 180 min.

After cooling of the digest, about 15 mL of de-ionized water was added to dissolve the precipitates formed on cooling and to dilute left over acid so that to minimize the dissolution of a filter paper by the digest residue while filtrating with Whatman, (110 mm, dia), filter paper. The round bottom flask was rinsed by using 8 mL de-ionized water two times and filtered. The filtrate was filled to 50 mL with de-ionized water. Triplicate digestions were carried out for each sample of soil and fennel fruit. Six blank solutions were prepared by digesting the mixture of reagents following the same procedure. The digested samples were kept in the refrigerator, until the level of all the elements in the sample solutions were determined by FAAS.

### Instrument calibration

Six points calibration curve were established by running series of the prepared working standard solutions. Correlation coefficients obtained for calibration curve were > 0.999. Immediately after calibration, the sample solutions were aspirated into the FAAS instrument and direct readings of the elemental concentrations was recorded. Calibration of the instrument was checked periodically during operation. Three replicate determinations were carried out on each sample. Blank analysis was also done similarly.

### Method validation

The method detection limit of each element was calculated as tree times the standard deviation of the blank (3σ_blank_, n = 6). The method detection limit for fennel fruit and soil samples are presented in Table 4. The method detection limits are low enough which clearly indicate that the selected elements can be detected at trace level by the FAAS method used.

**Table 4:** **Method detection limits for elements (for all metals, n = 6)**

Element	Ca	Mg	Mn	Fe	Zn	Cu	Cr	Co	Ni	Cd	Pb
**MDL for fennel fruit (μg/g)**	3	0.4	0.5	0.2	0.6	0.5	3	4	1	0.1	5
**MDL for soil (μg/g)**	5	0.5	1.5	0.4	2	2	3	3	3	1	5

MDL = Method detection limit.

To check the accuracy of digestion procedure and efficiency of the FAAS instrument spiked samples were prepared by adding a small known quantity of elemental standard solutions to both the soil and fennel fruit by applying similar digestion procedure, analysing for the levels of elements and calculating the recovery percent. The obtained percentage recovery for the fennel fruit varied from 93% to 108% and for the soil from 93% to 110% which are in the acceptable range. The reproducibility of the analytical procedure was checked by carrying out a triplicate analysis and calculating the coefficient of variation of the mean for each metal. In almost all cases, triplicate results did not differ by more than 10% of the mean.

### Statistical analysis

The multivariate analysis of variance for the equality of means and correlation between the elements in fennel fruit and soil samples were done using the SPSS 16.0.

## Results

The fennel fruit and the soil samples on which the plant was grown were analyzed for major (Ca, Mg, Fe, and Mn), trace essential (Cu, Zn, Cr and Co), and trace non-essential elements (Ni, Cd and Pb) with FAAS. The mean concentrations along with standard deviation of triplicate analysis are given from Tables 5 and 6 for the fennel fruit and soil samples, respectively.

**Table 5:** **Average concentration of elements (μg/g dry weight) in the fennel fruit samples from the four sites (mean ± SD, n = 3)**

Element	Ca	Mg	Fe	Mn	Cu	Cr	Co	Zn	Ni	Cd	Pb
**AA**	20,500 ± 290	2,390 ± 68	1,140 ± 67	45.8 ± 1.1	103 ± 4	97.7 ± 2.9	67.7 ± 3.7	37.1 ± 2.0	18.7 ± 0.5	1.90 ± 0.10	ND
**B**	23,000 ± 240	1,970 ± 83	1,900 ± 81	51.4 ± 1.3	48.4 ± 0.6	91.4 ± 2.4	26.2 ± 0.3	37.8 ± 2.2	24.2 ± 1.1	1.67 ± 0.10	ND
**DD**	21,400 ± 100	3,460 ± 93	1,480 ± 62	48.1 ± 6.2	62.5 ± 0.3	94.0 ± 4.7	49.5 ± 0.9	44.7 ± 2.5	24.0 ± 0.7	1.89 ± 0.08	ND
**FS**	22,900 ± 570	1,310 ± 58	1,470 ± 74	30.6 ± 0.7	23.9 ± 0.2	90.9 ± 0.4	70.8 ± 0.6	40.8 ± 1.3	24.2 ± 0.6	1.59 ± 0.10	ND

AA = Addis Ababa, B = Bichena, DD = Debre Markos, FS = Finote Selam and ND = not detected.

**Table 6:** **Average concentration of elements (μg/g dry weight) in the soil of the four sites (mean ± SD, n = 3)**

Element	Ca	Mg	Fe	Mn	Cu	Cr	Co	Zn	Ni	Cd	Pb
**AA**	1,440 ± 37	2,870 ± 59	27,600 ± 570	1,930 ± 30	101 ± 6	141 ± 5	143 ± 4	101 ± 3	98 ± 4	2.10 ± 0.10	ND
**B**	1,780 ± 37	2,260 ± 22	27,200 ± 180	1,980 ± 15	51.0 ± 0.5	127 ± 6	54.3 ± 0.7	99.0 ± 2	100 ± 1	2.90 ± 0.03	ND
**DD**	1,630 ± 76	3,310 ± 94	26,900 ± 170	1,980 ± 23	67.9 ± 2.1	129 ± 6	115 ± 1	104 ± 4	130 ± 2	2.41 ± 0.20	ND
**FS**	1,770 ± 41	1,260 ± 50	28,000 ± 75	1,460 ± 36	69.6 ± 0.6	129 ± 6	133 ± 7	103 ± 2	161 ± 1	1.66 ± 0.10	ND

AA = Addis Ababa, B = Bichena, DD = Debre Markos, FS = Finote Selam and ND = not detected.

Ten elements (Ca, Mg, Fe, Mn, Cu, Cr, Co, Zn, Ni, and Cd) were detected in both the fennel fruit and soil samples while Pb was not detected in both the fennel fruit and soil samples. The levels of elements however differ significantly among each other and to some extent between sampling sites. The variation between the sampling sites is due to variation in geological, geographical and climatic conditions of the sites. The trend of variation in fennel fruit sample in Addis Ababa site is Ca > Mg > Fe > Cu > Cr > Co > Mn > Zn > Ni > Cd, in Bichena site is Ca > Mg > Fe > Cr > Mn > Cu > Zn > Co > Ni > Cd, in Debre Markos site is Ca > Mg > Fe > Cr > Cu > Co > Mn > Zn > Ni > Cd and in Finote Selam site is Ca > Fe > Mg > Cr > Co > Zn > Mn > Ni > Cu > Cd. The trend of variation in soil sample in Addis Ababa site is Fe > Mg > Mn > Ca > Co > Cr > Cu = Zn > Ni > Cd, in Bichena site is Fe > Mg > Mn > Ca > Cr > Ni > Zn > Co > Cu > Cd, in Debre Markos site is Fe > Mg > Mn > Ca > Ni > Cr > Co > Zn > Cu > Cd and in Finote Selam site is Fe > Ca > Mn > Mg > Ni > Co > Cr > Zn > Cu > Cd. In all the cases concentration of Ca and Fe are much higher than the other elements in the both the fennel fruit and soil, respectively. In both the fennel fruit and the soil the level of Cd is much low at trace level but its concentration in the soil is relatively higher than the fennel fruit.

Table 5 indicates that except Cd which has lowest concentration in samples from Finote Selam and Ni in samples from Addis Ababa all the other essential trace elements lowest concentrations were found in samples from Bichena. All the trace elements have higher concentrations in samples from Addis Ababa (Cu, Cr, Co and Cd) except Zn in samples from Debre Markos and Ni in samples from Finote Selam. These go with the expectation of minor pollution in the capital city, Addis Ababa. Table 6 indicates that Co and Cr was the most accumulated trace element in the soil sample while the level of Cd was the lowest among the elements. Since Ethiopia is 10^th^ in the world and 1^st^ in Africa in cattle, leather industries are flourishing in the country. Relatively larger accumulation of Cr in the soil may be due to release of Cr in the nearby leather industries but the source of Co is unknown.

## Discussion

Elements are persistent in the environment and tend to bioaccumulate in plants and organisms. Bioaccumulation is of course a normal and essential process enabling the organism to have reserve for latter use for metalloproteins or cofactors or protect themselves against toxic effects (Kitata and Chandravanshi 2012). In this study the bioaccumulation of both trace and major elements were attempted to be verified by roughly comparing their concentration in one of the growth media, soil and those in plants.

**Figure 1:**
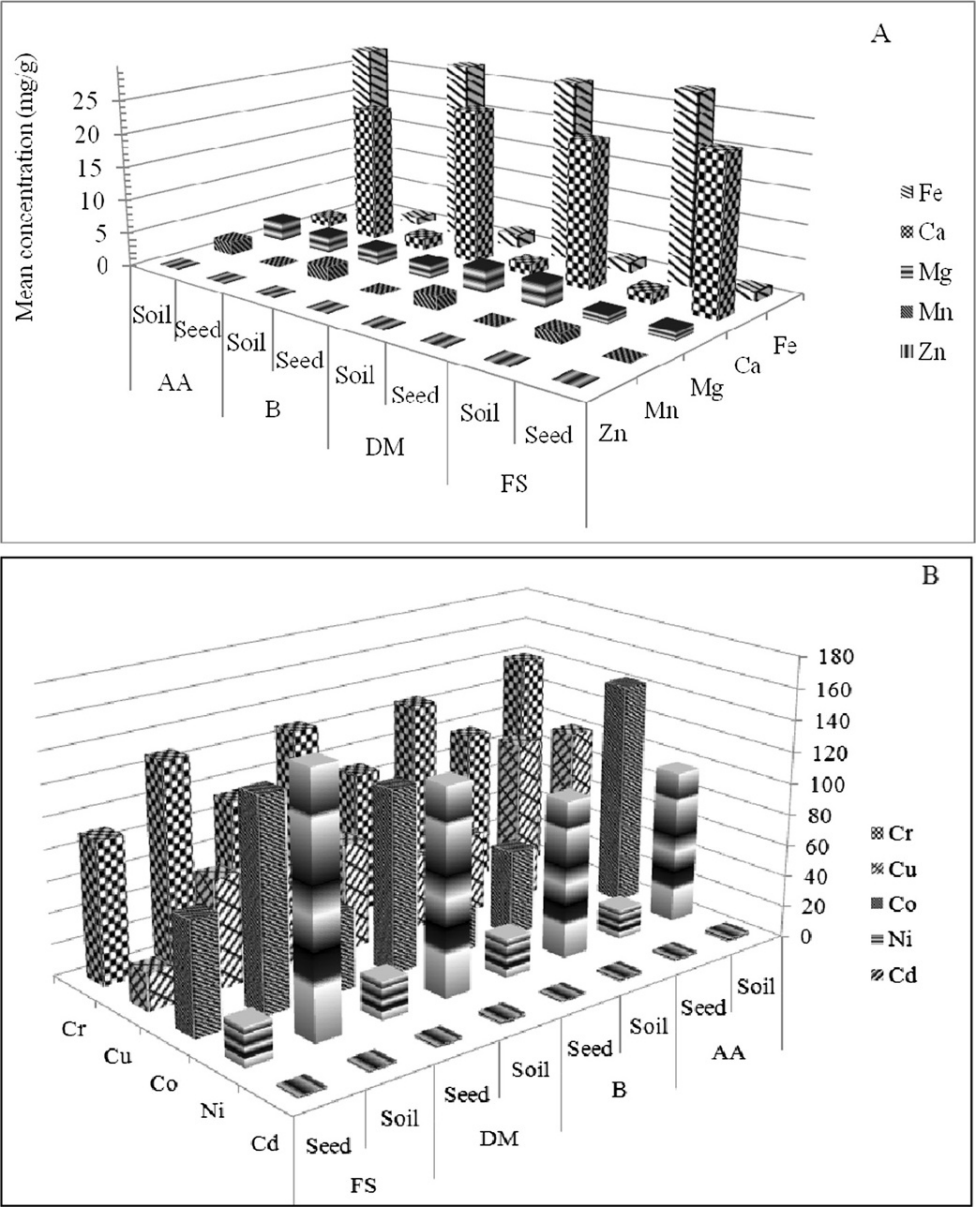
**Comparison of concentration of elements in the fennel fruit to elements in the soil. A**. Major elements and **B**. trace elements. AA = Addis Ababa, B = Bichena, DD = Debre Markos and FS = Finote Selam.

In comparison between soil and fennel fruit sample except for Ca fennel fruit showed less or comparable accumulation of elements found in the soil. Higher accumulation was observed for Ca only (Table 7 and Figure 1A). This may be because it is a seasonal plant and the soil is acidic. Ca accumulation ability of fennel fruit makes it valuable Ca resource for infants, pregnant women and elderly persons who need it very much in addition to its medicinal and spicy nature. However, it is not possible to estimate the daily intake of Ca from fennel fruit by infant, pregnant women and elderly persons living in different parts of the country due to their variable daily intake. Also there is no systematic data available on the daily intake of fennel fruit. For Mg and Cu fennel fruit showed comparable accumulation to that of the soil but for Fe, Mn, Cr, Co, Zn, Ni and Cd it showed low accumulation than available in the soil in all the sites. This might indicates that they are not found in bio-available form or fennel fruit has poor accumulation for these elements. The very low concentration of Cd and not detectable Pb in the fennel fruit indicate that the risk of toxicity of these elements is low when consuming it in row or processed forms. The FAO/WHO dietary recommendations of some of the metals are given in Table 8.

**Table 7:** **Accumulation coefficient of elements from the soil to fennel fruit**

Element	Sampling sites			
	AA	B	DM	FS
**Ca**	14.2	12.9	13.1	12.9
**Mg**	0.72	0.87	1.2	0.96
**Mn**	0.021	0.015	0.056	0.035
**Fe**	0.041	0.070	0.055	0.052
**Zn**	0.35	0.38	0.43	0.40
**Cu**	1.0	1.4	0.35	0.26
**Cr**	0.67	0.46	0.76	0.40
**Co**	0.49	0.19	0.11	0.54
**Ni**	0.25	0.19	0.19	0.15
**Cd**	0.91	0.58	0.78	0.96

AA = Addis Ababa, B = Bichena, DD = Debre Markos and FS = Finote Selam.

**Table 8:** **FAO/WHO dietary recommendation of some of the minerals (mg/day)**

Metals	Population groups			
	Infants	Pregnant women	Lactating women	Elderly (>65 year)
**Ca**	300-400	1200	1000	1300
**Mg**	26-36	220	270	190-230
**Zn**	2.8-4.1	5.5-10	7.2-9.5	4.9-7.0
**Fe**	8-9	1040	15	11-14

N.B. WHO recommendation for toxic metals in herbal medicines and products are: 10 mg/kg and 0.3 mg/kg for Pb and Cd, respectively.

The accumulation factor of the fennel fruit is evaluated and the result is presented in Table 7. Ca has highest accumulation coefficient (12.9–14.2) in all the sites. The reason for higher concentration of Ca in the fennel fruit is due to the acidity of the soil as a result of higher concentration of Fe (28,000–26,900 μg/g). The least accumulation is seen for Mn (0.015–0.056) in all the sites. Elements like Mg, Cu and Cd have variable concentration in the plant as that in the soil. For Cr, moderate difference in concentration is observed. In the case of Zn and Co, the concentration in the soil is higher than the fennel fruit at roughly by half. For other elements like Fe, Mn and Ni the concentration of the soil is very high (Figure 1A and B).

### Comparison of levels of elemental in fennel fruit with literature values

The major elements (Ca, Mg, Fe and Mn) have been determined in the fennel fruit in different countries from different points of view (nutritional, health problems, crop yield, etc.). Some of the available studies are presented in Table 9 along with the present study for comparison.

**Table 9:** **Summary of elemental levels reported fennel fruit worldwide (all values are in μg/g)**

Country	Ca	Mg	Fe	Mn	Ref.
**India**	23,900 ± 1300	5,110 ± 420	744 ± 20	72.3 ± 3.0	(Kumar et al. 2005)
**Turkey**	10,800 ± 220	2,770 ± 39	225 ± 9	27.8 ± 0.4	(Başgel & Erdemoğlu 2006)
**Turkey**	6,750 ± 120	3,400 ± 380	316 ± 35	33.4 ± 5.3	(Ozcan and Akbulut 2007)
**Pakistan**	-	-	1030 ± 290	877 ± 85	(Khattak and Khattak 2011)
**Ethiopia**	21,900 ± 1,190	2,280 ± 900	1,500 ± 310	44.0 ± 9.2	Present study

The level of Ca, in this study is comparable with the study from India (Kumar et al. 2005) but much higher than from Turkey (Başgel and Erdemoğlu 2006; Ozcan and Akbulut 2007). The level of Mg in this study is lower than from Turkey (Başgel and Erdemoğlu 2006; Ozcan and Akbulut 2007), and much lower than study from India (Kumar et al. 2005). The level of Fe in this study is much higher than studies from India and Turkey (Başgel & Erdemoğlu 2006; Ozcan and Akbulut 2007; Kumar et al. 2005). The level of Mn in this study is lower than study from India but higher than Turkey (Başgel and Erdemoğlu 2006; Ozcan and Akbulut 2007; Kumar et al. 2005). Generally the values are acceptable except Fe which is very high than the literature values.

Comparisons of levels of trace elements in fennel fruit reported in various parts of the world are summarized in Table 10. Results for Cu, Cr, and Co of this study are much higher than the literature values given in Table 10. This variation may be due to difference in the geographical location, climate, agricultural chemicals like fertilizers or errors in sample preparation and analysis. Extremely higher values for Cr may be related to pollution from the tannery industry found around the sampling areas. Results of this study for Zn, Ni and Cd are comparable with literature values.

**Table 10:** **Summary of trace elemental levels in fennel fruit worldwide (all values in μg/g)**

Country	Cu	Cr	Co	Zn	Ni	Cd	Ref.
**India**	10*.*9 ± 0.8	1.50 ± 0.35	0.54 ± 0.10	34.5 ± 4.4	1.46 ± 3.43	0.086 ± 0.034	(Kumar et al. 2005)
**Turkey**	16.2 ± 0.4	1.04 ± 0.20	0.40 ± 0.02	37.0 ± 2.4	5.40 ± 0.14	0.004 ± 0.001	(Başgel & Erdemoğlu 2006)
**Turkey**	8.28 ± 0.85	35.9 ± 9.12	-	20.8 ± 4.78	28.7 ± 1.38	0.50 ± 0.1	(Ozcan and Akbulut 2007)
**Pakistan**	117 ± 22	-	-	37.5 ± 3.0	-	-	(Khattak and Khattak 2011)
**Pakistan**				24.7 ± 0.5			(Chowdhary et al. 2014)
**Ethiopia**	59.5 ± 33.1	93.5 ± 3.1	53.5 ± 20.5	40.1 ± 3.5	22.8 ± 2.7	1.54 ± 0.16	Present study

### Multivariate analysis of variance

Variation in the mean levels of elements between the fennel fruit and soil samples were tested based on Tukey HSD multiple comparison model. Mg and Cd show significant differences (p < 0.05) at 95% confidence levels for all sampling sites whereas Zn in all sites and Fe except Addis Ababa and Debre Markos sites showed significant similarity (p > 0.05). These may indicate that fennel fruit has special storing capability for Mg and Cd irrespective of their concentration in the soil whereas Fe and Zn accumulation is the reflection of their concentration in the soil. Other elements show partial significant similarity and difference.

Ca (only in Bichena and Finote Selam), Cu (only in Finote Selam and Debre Markos), Co (only in Addis Ababa and Finote Selam) and Ni (only in Addis Ababa and Bichena) fennel fruit and soil showed significant similarity whereas for the other they showed significant difference. On the other hand, Mn and Cr showed half-half significant similarity and difference.

### Pearson correlation of elements

The high association between elements, evidenced by high positive correlation coefficient, can arise from common anthropogenic or natural sources as well as from similarity in chemical properties. The Pearson correlation matrices using correlation coefficient (r) for the fennel fruit samples of the four sits are shown in Table 11. There is poor negative relation between Ca and Mn, Mg and Fe, Mg and Cu, Mg and Cr, Mg and Ni, Mg and Cd, Mn and Zn, Fe and Zn, Fe and Ni, Zn and Cu, Zn and Cr, Cu and Ni, Cr and Co and Cr and Cd. Poor positive correlation was found between Ca and Fe, Zn, Co, Ni, and Cd; Mn and Cu, Cr, Co and Cd; Fe and Cd; Cu and Co and Cd; and Ni and Cr and Co. Mg has no poor positive relationship with any of the elements. Strong negative relation was seen between Ca and Cr; Mg and Mn and Co; Mn and Ni; Fe and Cr; Zn and Co, Ni and Cd; and Cu and Cr. Strong positive relationship found between Ca and Mg and Cu; Mg and Zn; Mn and Fe; Fe and Cu and Co; and Cd and Co and Ni.

**Table 11:** **Single tailed Pearson Correlation of elements in the fennel fruit (N = 12)**

Elements	Ca	Mg	Mn	Fe	Zn	Cu	Cr	Co	Ni	Cd
**Ca**	1.000	0.553^▲^	**−0.268**	0.402^**●**^	0.232^**●**^	0.588^▲^	−0.964	0.192^**●**^	0.118^**●**^	0.240^**●**^
**Mg**	0.553	1.000	−0.605	**−0.313**	0.768^▲^	**−0.047**	**−0.427**	−0.652	**−0.152**	−0.521
**Mn**	−0.268	−0.605	1.000	0.759^▲^	**−0.173**	0.442^**●**^	0.005^**●**^	0.345^**●**^	−0.587	0.185^**●**^
**Fe**	0.402	−0.313	0.759	1.000	**−0.154**	0.820^▲^	−0.617	0.585^▲^	**−0.352**	0.467^**●**^
**Zn**	0.232	0.768	−0.173	−0.154	1.000	**−0.048**	**−0.224**	−0.825	−0.663	−0.801
**Cu**	0.588	−0.047	0.442	0.820	−0.048	1.000	**−0.735**	0.410^**●**^	**−0.280**	0.312^**●**^
**Cr**	−0.964	−0.427	0.005	−0.617	−0.224	−0.735	1.000	**−0.266**	0.064^**●**^	**−0.265**
**Co**	0.192	−0.652	0.345	0.585	−0.825	0.410	−0.266	1.000	0.490^**●**^	0.941^▲^
**Ni**	0.118	−0.152	−0.587	−0.352	−0.663	−0.280	0.064	0.490	1.000	0.604^▲^
**Cd**	0.240	−0.521	0.185	0.467	−0.801	0.312	−0.265	0.941	0.604	1.0

AA = Addis Ababa, B = Bichena, DD = Debre Markos, FS = Finote Selam.

Bold font indicates poor negative relation (r > −0.500).

The ^**●**^ indicate poor positive relation (r < 0.500).

The ^▲^ indicate strong positive relation (r > 0.500).

The Pearson correlation matrices using correlation coefficient (r) for the fennel fruit and soil samples of the four sits are shown in Table 12. There is poor relation for Mg, Cu, Cr, and Co in Addis Ababa site, Ca, Fe, Co and Ni in Bichena site and Ni and Cd in Debre Markos site between elements in the fennel fruit and soil. There is no relation between Mg in the soil and fennel fruit in Bichena site which might imply different sources. The rest elements have good relation (at least greater than ± 0.5) between elements in the fennel fruit and soil.

**Table 12:** **Correlation of elements in the fennel fruit and soil samples in the four sits (n = 12)**

Site/Elements/	Ca	Mg	Fe	Mn	Cu	Cr	Co	Zn	Ni	Cd
**AA**	−0.842	0.425^**●**^	−0.663	0.985^▲^	**−0.290**	0.464^**●**^	0.300^**●**^	−0.993	0.683^▲^	0.500^▲^
**B**	**−0.395**	0.000*	**−0.092**	0.947^▲^	0.615^▲^	0.957^▲^	**−0.366**	0.704^▲^	**−0.211**	−0.500
**DM**	−0.613	−0.707	0.979^▲^	0.756^▲^	0.936^▲^	−0.699	0.977^▲^	−0.998	0.219^**●**^	**−0.013**
**FS**	−0.721	−0.689	0.569^▲^	−0.966	−0.693	−0.950	−0.500	0.862^▲^	−0.984	−0.619

AA = Addis Ababa, B = Bichena, DD = Debre Markos, FS = Finote Selam.

Bold font indicates poor relation (r > − 0.500).

The * indicate no relation.

The ^**●**^ indicate poor positive relation (r < 0.500).

The ^▲^ indicate strong positive relation (r > 0.500).

## Conclusion

In this study fennel fruit and the soil in which the plant was grown was analyzed for major and trace elements (Ca, Mg, Fe, Mn, Cu, Cr, Co, Zn, Ni, Cd and Pb). The fennel fruit was found to have higher concentration of Ca, moderate concentration of Mg and Fe, and lower but considerable concentration of Cr, Cu, Co, Mn and Zn; relatively low concentration of Ni and very low concentration of Cd. The soil samples contain the highest concentration of Fe followed by Mg, Ca Mn, Co, Cr, Zn, Ni, and Cu. From the trace elements Cu showed higher accumulation factor. The concentration of Cd was higher in the soil than in the fennel fruit. Concentration of Pb in both the soil and the fennel fruit was below the method detection limit. For all the elements higher or comparable concentration were found in the soil and the fennel fruit except Ca in all the sites and Cu in Addis Ababa and Bichena sites.

The Ethiopian fennel fruit contain higher amount of Ca, and considerable amount of other micronutrients. It contains only trace level of Cd and Pb was not detected which showed that it is safe for human consumption in daily life. These results may also indicate that fennel plant has lesser tendency to accumulate toxic elements in the fruit.
